# Regulated expression and function of the GABA_B_ receptor in human pancreatic beta cell line and islets

**DOI:** 10.1038/s41598-020-69758-6

**Published:** 2020-08-10

**Authors:** Latif Rachdi, Alicia Maugein, Severine Pechberty, Mathieu Armanet, Juliette Hamroune, Philippe Ravassard, Stefano Marullo, Olivier Albagli, Raphael Scharfmann

**Affiliations:** 1 Institut Cochin, INSERM U1016, CNRS UMR 8104, Université de Paris, 123 bd du Port-Royal, 75014 Paris, France; 2 Assistance Publique Hôpitaux de Paris, Cell Therapy Unit, Saint Louis Hospital, 75010 Paris, France; 3 Paris Brain institute (ICM), INSERM U1127, CNRS UMR 7225, Sorbonne Université, 75013 Paris, France

**Keywords:** Cell signalling, Diabetes, Multihormonal system disorders

## Abstract

G protein-coupled receptors are seven transmembrane signaling molecules that are involved in a wide variety of physiological processes. They constitute a large protein family of receptors with almost 300 members detected in human pancreatic islet preparations. However, the functional role of these receptors in pancreatic islets is unknown in most cases. We generated a new stable human beta cell line from neonatal pancreas. This cell line, named ECN90 expresses both subunits (*GABBR1* and *GABBR2*) of the metabotropic GABA_B_ receptor compared to human islet. In ECN90 cells, baclofen, a specific GABA_B_ receptor agonist, inhibits cAMP signaling causing decreased expression of beta cell-specific genes such as *MAFA* and *PCSK1,* and reduced insulin secretion. We next demonstrated that in primary human islets, *GABBR2* mRNA expression is strongly induced under cAMP signaling, while *GABBR1* mRNA is constitutively expressed. We also found that induction and activation of the GABA_B_ receptor in human islets modulates insulin secretion.

## Introduction

Type 2 diabetes mellitus (T2DM) is the most common metabolic disease worldwide, affecting more than 350 million people. It is a multigenic disease showing increased insulin resistance progressively weakening pancreatic beta cell response. In patients with T2DM, both beta cell function and beta cell mass are decreased. Thus, understanding the regulation of beta cell function is critical to identify mechanisms underlying the development of T2DM^1^.

G protein-coupled receptors (GPCRs), which modulate a variety of physiological responses, are potential targets for anti-diabetic compounds^2^. These seven transmembrane receptors are coupled to heterotrimeric G proteins, such as Gα_s_, Gα_i/o_, Gα_q/11_ and Gα_12/13_. GPCR coupling to Gα_s_ stimulates adenylyl cyclase, which increases cyclic AMP levels while coupling through Gα_i/o_ inhibits adenylyl cyclase activation^3,4^.

Gamma aminobutyric acid (GABA) is an inhibitory neurotransmitter that acts in an autocrine and/or paracrine manner by activating GABA_A_ and GABA_B_ receptors at the plasma membrane. The GABA_A_ receptor is a five-subunits chloride ion channel whereas the GABA_B_ receptor (also known as the metabotropic receptor) is a GPCR heterodimer composed of two subunits, GABBR1 and GABBR2. The GABBR1 subunit binds GABA, whereas the GABBR2 subunit is responsible for Gα_i/o_-protein-coupled activation, leading to inhibition of adenylyl cyclase and consequently, to decreased cAMP signaling. Previous studies demonstrated that the presence of both subunits is necessary for GABA-induced signaling in individual cells^5,6^. GABA plays major roles in the brain. Interestingly, beta cells express glutamic acid decarboxylase (GAD1/GAD67 in mice and GAD2/GAD65 in human) the enzyme involved in the synthesis of GABA from glutamate^7^. However, how GABA signals in islets and particularly in human beta cells has not been fully explored. Whereas the expression of GABA_A_ receptors in human islets and their altered expression and sensitivity in islets from T2DM patients has been reported^6,8,9^, GABA_B_ receptor mediated signaling in human beta cells remains controversial^10–13^.

Here, we investigated GABA_B_ receptor signaling in human beta cells using in a newly developed human beta cell line, ECN90 cells. Both GABBR1 and GABBR2 subunits are expressed in these cells. By using baclofen, a specific synthetic agonist of the GABA_B_ receptor^14^, we demonstrated that the GABA_B_ receptor is functional and modulates beta cell differentiation and insulin secretion in ECN90 cells. We next demonstrated that human islets do not express functional GABA_B_ receptors. We observed that while *GABBR1* mRNA is expressed in a constitutive fashion in human islets, induction of *GABBR2* expression requires activation of the cAMP signaling to give rise to functional GABA_B_ receptors. Our data also indicated that signaling through the GABA_B_ receptor is a previously unknown feedback mechanism regulating human beta cell differentiation and function.

## Results

### ECN90: a beta cell line derived from human neonatal pancreas

The ECN90 cell line was derived from a fragment of pancreas from a 4-months old patient suffering of hyperinsulinemic hypoglycemia of infancy (PHHI). A protocol similar to the one previously developed to generate human beta cell lines from fetal pancreas was used^15^. Briefly, the free margin of the neonatal pancreatic tissue was simultaneously transduced with 2 lentiviral vectors expressing SV40T and hTERT both under the control of the rat insulin2 promoter and then transplanted under the kidney capsule of immune-incompetent SCID mice. Three months following transplantation, immunostainings indicated the presence of INSULIN^+^/SV40T^+^cell clusters with a fraction of INSULIN^+^ cells that proliferated, as shown by Ki67 staining (Fig. S1). Seven months post-transplantation, we observed large insulinomas positive for INSULIN, SV40T and Ki67 (Fig. S1). From serial transplantations ^16^, we derived a cell line we named ECN90 (Fig. S2A) that stained positive for INSULIN, for PDX1, a transcription factor mainly expressed in beta cells, for SV40T and Ki67 (Fig. S2B,C).

### ECN90 expresses both subunits of the metabotropic GABA_B_ receptor

**Figure 1:**
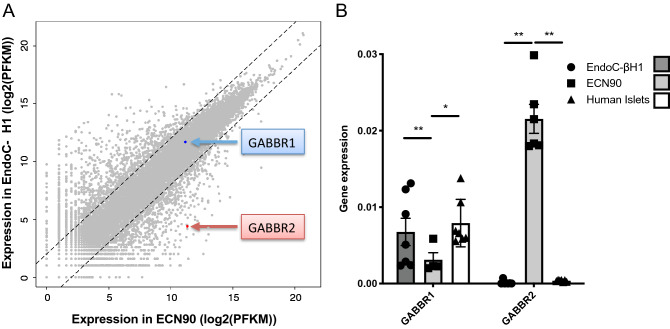
Expression *GABBR1* and *GABBR2* in human beta cell lines and human islets. (**A**) Scatterplot illustrating the comparative RNAseq analyses of 2 human beta cell lines EndoC-βH1 and ECN90. Blue and red arrows highlight *GABBR1* and *GABBR2* mRNA levels. (**B**) Expression of *GABBR1* and *GABBR2* mRNA by RT-qPCR in EndoC-βH1, ECN90 and human islets. Data are shown as the mean ± SEM; *n* = 6. *P < 0.05; **P < 0.01 relative to control by Student’s *t* test.

Comparative RNAseq analyses were performed between ECN90 cells and the previously developed EndoC-βH1 cells. Expression profiles are depicted in Fig. 1A as scatter plots. Most of the transcripts are expressed at remarkably similar level in both cell lines, further indicating the beta cell identity of ECN90 cells. Both lines expressed *GABBR1* at similar levels, whereas the expression *of GABBR2* was more than 100 times higher in ECN90 cells compared to EndoC-βH1 cells. RT-qPCR analyses further indicated that *GABBR1* was expressed in ECN90 cells, EndoC-βH1 cells and human islets. On the other hand, *GABBR2* was only detected in ECN90 cells (Fig. 1B).

**Figure 2:**
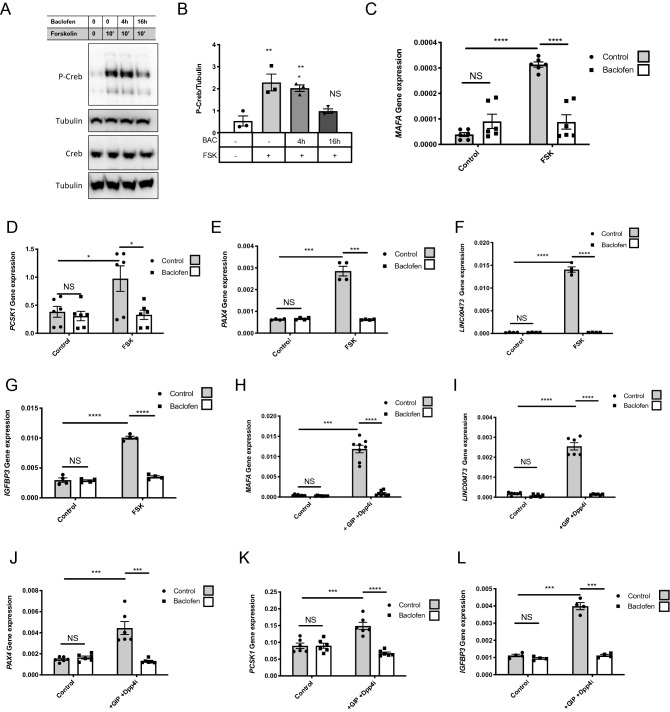
Effects of baclofen treatment on ECN90. (**A,B**) Western blot and quantification of P-CREB (*n* = 3) in ECN90 pretreated during 0-16 h with Baclofen (BAC) and next pulsed during 10′ with forskolin (FSK). (**C–G**) ECN90 were pretreated with or without Baclofen during 16 h and next pulsed for 1 h with FSK. RT-qPCR analyses indicate that Baclofen treatment blunts the induction by FSK of *MAFA*, *PCSK1*, *PAX4*, *LINC00473* and *IGFBP3*. (**H–L**) ECN90 were pretreated with or without Baclofen during 16 h and next pulsed for 1 h with GIP and DPP4 inhibitor (used to inhibit GIP degradation). RT-qPCR analyses indicate that BAC treatment blunts the induction by GIP and DPP4 inhibitor for the same cAMP induced genes. Data are shown as the mean ± SEM (n = 3–6). *P < 0.05; **P < 0.01; ***P < 0.005; ****P < 0.001; NS = not significant relative to control by Student’s *t* test.

GABA_B_ receptor function in ECN90 cells was tested using its specific agonist, baclofen (BAC). As the GABA_B_ receptor is a Gα_i/o_-coupled receptor^17–19^, we activated the ECN90 cell adenylyl cyclase with forskolin and tested whether pretreatment with baclofen decreased such activation. Exposure of ECN90 to forskolin promoted the phosphorylation of CREB at Ser133 within 10 min while pretreatment (16 h, 100 µM) with baclofen inhibited CREB phosphorylation (Fig. 2A,B for quantification). To further investigate the function of the GABA_B_ receptor in ECN90, we searched for genes whose induction by forskolin would be blunted upon pretreatment with baclofen (16 h, 100 µM). Forskolin treatment induced a robust increase in *MAFA, PCSK1, PAX4, IGFBP3 and the non-coding long RNA Linc00473,* as previously shown in our microarray analyses from forskolin treated human EndoC-βH1 cells^20^. This induction was blunted upon pretreatment with baclofen (Fig. 2C–G). The repressive effect of baclofen on *MAFA, PCSK1, PAX4, Linc00473* induction by forskolin was reproduced when ECN90 were treated with the Gastric Inhibitory Polypeptide (GIP) (Fig. 2H–L), an incretin that is also an inducer of the cAMP pathway^21,22^.

As described above, ECN90 cells have been transformed using SV40T. To determine whether *GABBR2* expression is dependent of *SV40T* expression, we knocked-down *SV40T* using siRNA. *SV40T* depletion increased INSULIN staining and content (Fig. S3), *IAPP* and *CDKN1A* mRNA levels (Fig. S4A) as previously observed upon *SV40T* depletion in EndoC-βH1 cells^23^. Interestingly, SV40T knock-down did neither modify *GABBR1* and *GABBR2* mRNA levels, nor the ability of baclofen to inhibit the induction of MAFA by forskolin, indicating that *GABBR2* expression and GABA_B_ receptor function in ECN90 are independent of SV40T expression and beta cell immortalization (Fig. S4B).

### cAMP signaling *regulates GABBR2* expression and function in EndoC-βH1 cells and in human islets

**Figure 3:**
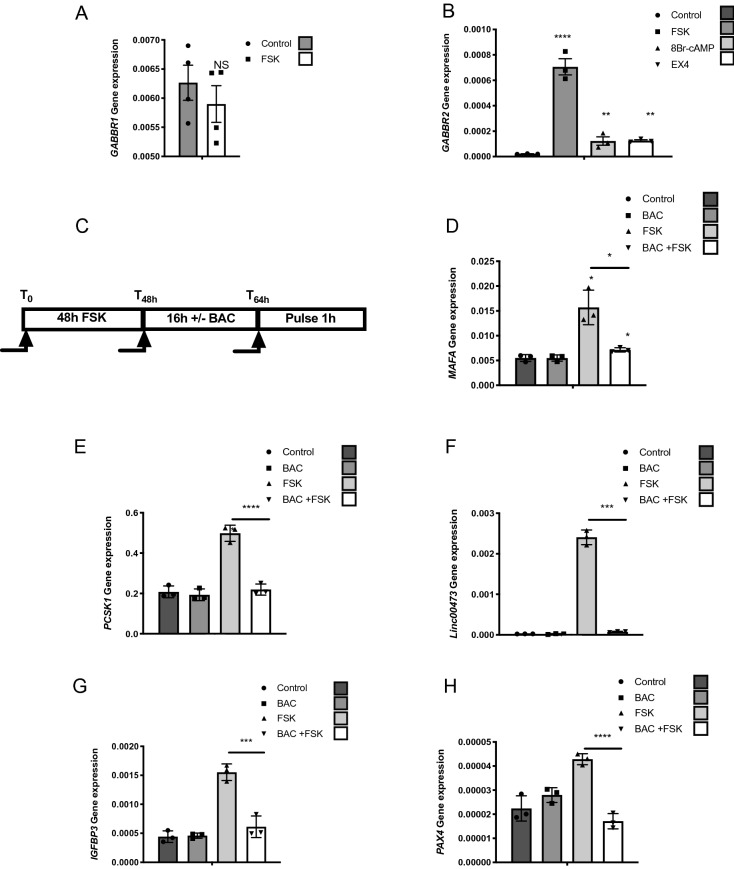
cAMP signaling induces *GABBR2* expression in EndoC-βH1. (**A**) RT-qPCR analyses of *GABBR1* mRNA in EndoC-βH1 treated with forskolin (FSK). (**B**) RT-qPCR analyses of *GABBR2* mRNA in EndoC-βH1 following 48 h treatment with DMSO (0.1%; control condition), FSK, 8Br-cAMP or exendin4 (EX4). (**C**) Schematic representation of the timeline for the conditioning pulse experiment. (**D–H**) EndoC-βH1 were preconditioned during 48 h with FSK to induce *GABBR2* expression. The medium was next changed and cells were further cultured during 16 h with or without Baclofen (BAC) and finally pulsed for 1 h with FSK. RT-qPCR analyses indicate that BAC treatment blunts *MAFA* induction by FSK. Result for *PCSK1*, *LINC00473, PAX4* and *IGFBP3* induction are also shown. Data are shown as the mean ± SEM (n = 3–4). *P < 0.05; ***P < 0.005; ****P < 0.001; NS = not significant relative to control by Student’s *t* test.

Basal *GABBR2* mRNA levels are extremely low both in EndoC-βH1 cells and in human islet preparations (Ct ~ 33–34 for cyclophilin at Ct ~ 20) (Fig. 1B). Moreover, GABA_B_ receptor was not functional in EndoC-βH1 cells and in human islets as demonstrated by the lack of repressive effect of baclofen on forskolin-induced *MAFA* and *PCSK1* induction (Fig. S5). Interestingly, mining our previous results from microarray analyses suggested that forskolin may increase *GABBR2* mRNA levels in EndoC-βH1 cells^20^. We validated this hypothesis by RT-qPCR that indicated that while a 48 h treatment with forskolin does not modify *GABBR1* expression (Fig. 3A), it robustly increased *GABBR2* mRNA levels in EndoC-βH1 cells (Fig. 3B). A similar induction of *GABBR2* was also observed upon treatments with either 8Br-cAMP or exendin4 (EX4) (Fig. 3B), 2 different activators of the cAMP pathway. Moreover, following *GABBR2* induction, baclofen treatment blunted forskolin-induced *MAFA* expression, indicating that the GABA_B_ receptor is functional under such conditions (Fig. 3C,D). The repressive effect of baclofen on *PCSK1, Linc00473, IGFBP3 and PAX4* induction by forskolin was also observed in EndoC-βH1 (Fig. 3E–H).

**Figure 4:**
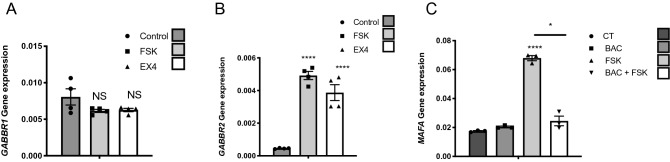
cAMP signaling induces *GABBR2* expression in human islets. (**A**) RT-qPCR analyses of *GABBR1* mRNA in human islets treated with forskolin (FSK) or exendin4 (EX4). (**B**) RT-qPCR analyses of *GABBR2* mRNA in human islets following 48 h treatment with DMSO (0.1%; control condition), FSK or EX4. (**C**) Human islets were preconditioned during 48 h with FSK to induce GABBR2 expression. The medium was next changed and cells were further cultured during 16 h with or without Baclofen (BAC) and finally pulsed for 1 h with FSK. RT-qPCR analyses indicate that under such conditions, BAC treatment blunts *MAFA* induction by FSK. Data are shown as the mean ± SEM (n = 3–4). *P < 0.05; ****P < 0.001; NS = not significant relative to control by Student’s *t* test.

Based on this finding in EndoC-βH1 cells, we examined the cAMP dependent induction of *GABBR2* on preparations of human islets. Forskolin and exendin4 (48 h treatments) did not modify *GABBR1* expression (Fig. 4A), while, they both increased *GABBR2* expression (Fig. 4B). As in EndoC-βH1 cells, under such conditions, GABA_B_ receptor was functional, as demonstrated by the repressive effect of baclofen on forskolin-induced *MAFA* expression in human islets (Fig. 4C).

### Signaling through the GABA_B_ receptor limits the induction of insulin secretion by cAMP

**Figure 5:**
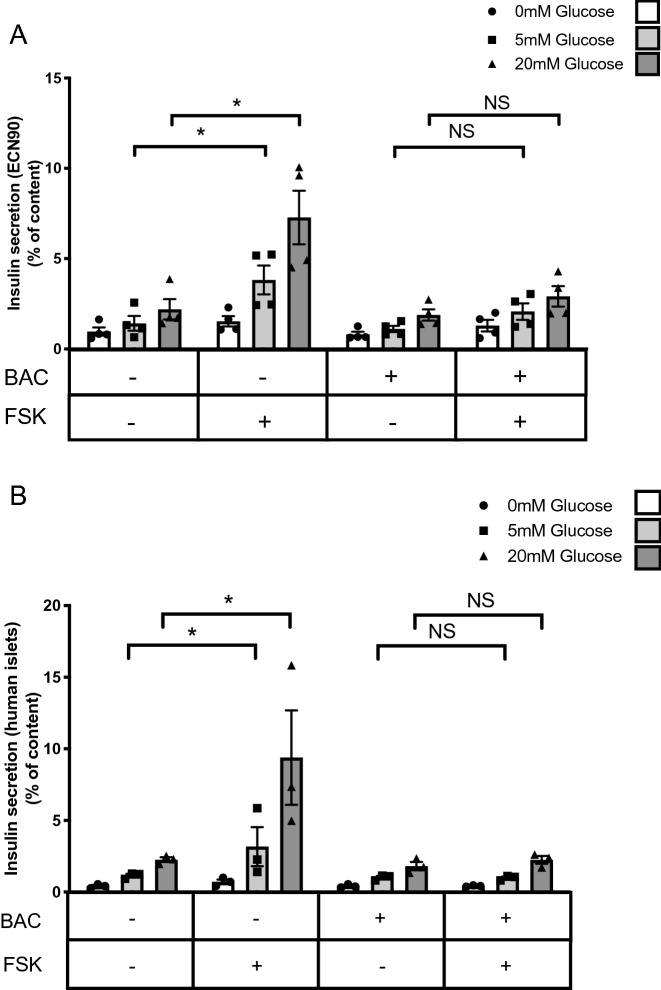
Baclofen treatment blunts forskolin-induced insulin secretion. (**A**) ECN90 were pretreated with or without Baclofen (BAC) during 16 h and next pulsed for 1 h with or without forskolin (FSK) followed by a 40 min insulin secretion test at 0, 5 and 20 mM glucose. Secreted insulin is presented as % of content. (**B**) Human islets were preconditioned during 48 h with FSK to induce *GABBR2* expression. They were next pretreated with or without BAC during 16 h and next pulsed for 1 h with FSK followed by a 40 min insulin secretion test at 0, 5 and 20 mM glucose. Secreted insulin is presented as % of content. Data are shown as the mean ± SEM (n = 3–4). *P < 0.05; NS = not significant relative to control by Student’s *t* test.

We finally evaluated whether signals through the GABA_B_ receptor affect the beta cell function. We assessed the effect of baclofen treatment on glucose and forskolin stimulated insulin secretion in ECN90 cells. Forskolin induced insulin secretion in a glucose-dependent manner and this effect was blunted when ECN90 cells were pre-treated with baclofen (Fig. 5A). Similar experiments were performed using human islets that had been treated for 48 h with forskolin to induce GABBR2 expression. As in ECN90 cells, baclofen treatment blunted forskolin-induced insulin secretion of human islets (Fig. 5B).

## Discussion

Here we have developed a new human beta cell line from neonatal pancreas and demonstrated that both human beta cell lines and primary human islets express, in a tightly regulated fashion, functional GABA_B_ receptors that regulate insulin secretion.

We previously generated several functional human beta cell lines (EndoC-βH1 and EndoC-βH2) from human fetal pancreatic fragments^15,16^. In the present study, we generated an additional one, named ECN90. This line is interesting for a number of reasons: (i). We derived ECN90 from a fragment of neonatal pancreas. Thus, with our protocol of targeted oncogenesis, while human adult beta cells are resistant to transformation^16^, fetal ^15,16^ but also neonatal (the present study) human pancreases are permissive for the generation of beta cell lines; (ii) ECN90 cells carry the HLA-I haplotype: HLA-A*02:01/03:01, -B*40:01/49:01, -C*03:04/07:01. This cell line provides a unique tool to model in vitro beta cell death in type 1 diabetes by assaying the cytotoxic effects of CD8T cell clones against human beta cells^24^ and thus to progress in the definition of ways to protect human beta cells against destruction.

Here, we used ECN90 cells as a first model to study the expression and function of the GABA_B_ receptor in human beta cells. The GABA_B_ receptor is a Gα_i/o_-protein-coupled receptor composed of 2 subunits, namely GABBR1 and GABBR2 with both subunits necessary for signaling through this receptor^25–28^. The GABA_B_ receptor is mainly present and functional in the central and peripheral nervous system where both GABBR1 and GABBR2 subunits are present^29,30^. Outside the nervous system, GABBR1 was detected in some visceral tissues such as the stomach, intestine, heart, and spleen^31^, while *gabbr2* expression has been observed in mouse liver and islets^32^. We demonstrate here that ECN90 cells express both subunits of the GABA_B_ receptor. We also show that this receptor can be activated by baclofen, a specific synthetic agonist of the GABA_B_ receptor that does not interact with GABA_A_ receptors^5,14^. The expression in beta cells of the GABA_B_ receptor known to be highly enriched in neurons is not fully unexpected, in the view of the large number of similarities between neurons and beta cells^33^. ECN90 cells can thus now be used as a model system to study signaling through the GABA_B_ receptor in non-neural cells.

Our data indicate that under basal culture conditions, ECN90 cells express both GABBR1 and GABBR2, while EndoC-βH1, an independent human beta cell line and human islets express GABBR1 but not GABBR2. At that stage, we do not have clues to explain the differences from one cellular model to the other. However, it could be due to differences in the expression between ECN90 and EndoC-βH1 of transcription factors that regulate GABBR2 expression levels. But importantly, GABBR2 expression can be induced in EndoC-βH1 cells and in human islets upon treatment with compounds that increase the cAMP signaling pathway such as forskolin or exendin4, giving rise to functional GABA_B_ receptors. It is well established that signaling by the GABA_B_ receptor is a tightly-regulated process. However, while information is available on signals implicated in post-translational regulation of GABBR2 signaling, such as its trafficking^34^, or its phosphorylation by the Protein Kinase A^35^, information on its transcriptional regulation remains extremely scarce. For example, the mechanisms that explain the specificity of GABBR2 expression in the nervous system remain poorly defined. GABBR2 expression in human beta cells and its regulation by the cAMP pathway will represent an innovative model to progress on the mechanism that regulate its expression.

We demonstrate here that signaling through the GABA_B_ receptor inhibits the cAMP pathway, known to play major roles in beta cell. Through paracrine effects, cAMP signaling modulates glucose-stimulated insulin secretion^36^. Specifically, alpha cells release glucagon that signals on beta cells through the Gα_s_-coupled Glucagon receptor, increases cAMP levels and insulin secretion^37,38^. In parallel, delta cells secrete somatostatin that binds the G_i_-coupled somatostatin receptor 2 on beta cells, and decreases cAMP levels and insulin secretion^39^. Here, we demonstrate that similarly, activation of the G_i_-coupled GABA_B_ receptor decreases cAMP levels and insulin secretion. In this context, it is important to note that human beta cells express the enzyme GAD2 and thus produce GABA that is co-secreted with insulin^40^. GABA may thus act through an autocrine loop to bring insulin back to basal levels following stimulation, a major property of mature beta cells^8,9,41^.

The cAMP pathway is also important to maintain the beta cell differentiation status^42^ and signals through the GABA_B_ receptor counteract this process. Indeed, activating the cAMP pathways increased the expression of a number of genes important for beta cell function. It is the case for MAFA, a factor implicated in insulin gene transcription^43^ and for the proconvertase PCSK1, an enzyme implicated in the processing of proinsulin^44^. Forskolin treatment also increased PAX4 expression, a transcription factor essential for beta cell development during prenatal life^45^ that is also implicated in beta cell proliferation and protection against degeneration^46^. Finally, forskolin treatment induced the expression of the long non-coding RNA LINC00473 with yet unknown function in beta cells. This cAMP induction of LINC00473 was previously described in non-small cell lung cancer^47^. Interestingly, such inductive effects are blunted upon activation of the GABA_B_ receptor, which suggests a tight balance between positive and negative inducers of the cAMP pathway.

Antidiabetic medications such as incretins improve islet function through cAMP production. Here, we show that cAMP signaling induces functional GABA_B_ receptors that counteract incretin effects. A number of type 2 diabetic patients are insensitive to incretins without any clue^48^. Whether it is due to GABA_B_ receptors over-activation might be evaluated.

In conclusion, our data demonstrate that in human beta cells, signaling through the GABA_B_ receptor participates in an autocrine feedback inhibition loop that regulates beta cell specific gene expression and insulin secretion.

## Methods

### Ethical statement

This study was performed according to the Declaration of Helsinki and the Declaration of Istanbul. No tissues were procured from prisoners. As the French Biomedical Agency regulates the graft allocation system in France, every organ was allocated and approved by the ethics committee of the French Biomedical Agency to be in accordance with French laws. Neonatal tissue was collected with written informed consents from the parents and in compliance with French bioethic legislation certified by the French Biomedical Agency. Human Islet collection was approved by the ethics committee of the French Biomedical Agency. Experiments using human graft in mice were approved by the animal experimentation ethics committee of Paris Descartes University and Sorbonne University (Paris, France). Experiments using mice were certified by the Direction Departementale de la Protection des Populations for the French Ministry of Research, Health and Agriculture (Paris) under agreement number A75-13–19 in accordance with approved guideline of French and European legislation. Human Islet collection was certified by the French Biomedical Agency Guidelines and registered in the French Ministry of Health under the number PFS12-006.

### Derivation *from neonatal pancreas* of ECN90, a *human β cell line*

A fragment from neonatal pancreas was collected, cut into pieces, digested with type IV collagenase (Sigma-Aldrich) and transduced with lentiviral vectors. Two *loxP* sites flank the integrated sequences expressing SV40T and hTERT, allowing subsequent excision dependent on Cre recombinase expression^15^. The tissue was next transplanted under the kidney capsule of immune-incompetent SCID (Charles River, L’Arbresle, France) as described^49^. Following 3 successive rounds of transplantation, we derived the cell line ECN90 that is cultured at 37 °C in 5% CO_2_ in Advanced DMEM/F12 medium (Thermo Fisher Scientific) supplemented with 2% bovine serum albumin fraction V (Roche), 6.7 ng/ml sodium selenite, 10 mM nicotinamide (Calbiochem), 50 μM β-mercaptoethanol (Sigma-Aldrich) and penicillin/streptomycin (Thermo Fisher Scientific).

### Human islets

Human islets were provided by the Human islet core facility of St-Louis Hospital (APHP, France). They were obtained from pancreata of seven brain-dead donors (mean age 55.67 ± 4.68 years; BMI 25.4 ± 4.36 kg/m^2^) with signed informed consents according to the procedures approved by the French Agency of Biomedicine (Supplemental Table 1). Islets were isolated, handpicked and cultured 48 h in 12-well plates (50–100 islets per well) in CMRL medium supplemented with 10% fetal calf serum, Hepes and penicillin/streptomycin (all from Thermo Fisher Scientific).

### Cells and islets treatments

The following compounds were used for treatments of EndoC-βH1^16^, ECN90 and human islets: forskolin (FSK) (Tocris;10 μM); R-Baclofen (Tocris;100 µM), linagliptin (Dpp4i; Selleckchem; 100 nM) GIP (Tocris, 100 nM), exendin4 (Tocris, 5 nM), 8Br-cAMP (Tocris; 1 µM).

### Glucose-stimulated insulin secretion (GSIS)

ECN90 cells were seeded onto Matrigel/fibronectin-coated 12-well plates at 2.5 × 10^5^ cells/well and human islets were cultured in 12-well plates (50 islets per well in triplicates). They were both starved in DMEM (Thermo Fisher Scientific) containing 0.5 mM glucose for 24 h, washed twice and then preincubated in Krebs–Ringer bicarbonate Hepes buffer (KRBH) containing 0.2% BSA in the absence of glucose for 1 h. Insulin secretion was measured following a 40 min incubation with KRBH containing 0.2% BSA that contained varying glucose concentrations. Glucose stimulation was performed in the presence or absence of 10 μM FSK. For insulin content measurements, cells and islets were lysed in the culture wells in 50 mM Tris, pH 8.0, 1% Nonidet P-40, 0.5% sodium deoxicolate, 0.05% SDS, 100 mM NaCl, 5 mM EDTA (Thermo Fisher Scientific), and anti-protease tablets (Roche) for 20 min on ice. Insulin secretion and content were measured by ELISA (Mercodia AB, Uppsala, Sweden) as described^15^.

### RNA isolation, reverse transcription, and RT-qPCR

RNeasy Micro Kit (Qiagen) was used to extract total RNA from beta cell lines and human islets^50^. Genomic DNA was removed by DNAse treatment following the RNeasy Micro Kit protocol. Maxima First Strand cDNA Kit (Thermo Fisher Scientific) was used to synthesize cDNA. RT-qPCR was performed using Power SYBR Green mix (Applied Biosystems) with a QuantStudio 3 analyzer. The comparative method of relative quantification (2ddCT) was used to calculate the expression levels of each target gene, normalized to Cyclophilin-A transcript. Reactions with and without reverse transcriptase (RT + and RT−) were used as control for the absence of genomic DNA contamination. Reactions with and without reverse transcriptase (RT + and RT−) were used as control of no genomic DNA contamination as RT- cDNA sample generate no signal with cyclophilin primers, indicating complete elimination of gDNA. Custom primers were designed with Primer-Blast online, and their efficiency was determined for each with a serial dilution of cDNA samples. The list of primers is presented in Supplemental Table 2.

### RNA-seq: library preparation and analysis

RNA-seq was provided by our Genom’IC lab facility in Institut Cochin. RNA concentrations were measured using nanodrop (Thermo Fisher Scientific, USA). The quality of the RNA (RNA integrity number or RIN) was determined on the Agilent 2,100 Bioanalyzer (Agilent Technologies, Palo Alto, CA, USA). 800 ng of total RNA sample (RIN > 9) was processed to construct the libraries using TruSeq Stranded mRNA kit (Illumina). Libraries were quantified by RT-qPCR using the KAPA Library Quantification Kit for Illumina Libraries (KapaBiosystems, Wilmington, MA) and library profiles were assessed using the DNA High Sensitivity LabChip kit on an Agilent Bioanalyzer. Libraries were sequenced on an Illumina Nextseq 500 instrument using 75 base-lengths read V2 chemistry in a paired-end mode. After sequencing, a first analysis based on AOZAN software (ENS, Paris) was applied to demultiplex and control the quality of the raw data (based of FastQC modules / version 0.11.5). Obtained fastq files were then aligned using STAR algorithm (version 2.5.2b). Reads were then counted using Featurecount (version Rsubread 1.24.1) and the statistical analyses on the read counts were performed with the DESeq2 package version 1.14.1. RNA-seq data are available in the NCBI’s Gene Expression Omnibus (GEO) database (accession GSE155482).

### siRNA transfection

ECN90 cells were transfected using Lipofectamine RNAiMAX (Thermo Fisher Scientific) as previously described^50^. Custom *Silencer*® Select siRNA for SV40T^51^ (Thermo Fisher Scientific), or ON-TARGETplus nontargeting control pool (siCTRL) were used (Dharmacon, GE healthcare Life Sciences) at a final concentration of 80 nM. Briefly, siRNA and Lipofectamine RNAiMAX were combined in OptiMEM (Thermo Fisher Scientific) and applied to the cells. Medium was replaced 2.5 h later with fresh ECN90 culture medium^50^.

### Immunostainings

Immunohistochemistry and immunocytochemistry were performed as previously described^15^ using the following antibodies guinea pig anti-insulin antibody (1/500; A0564, DakoCytomation); rabbit anti-human PDX1 antibody (1/2,000) ^52^ mouse anti-SV40T (1/50; DP-02, Calbiochem Merck Biosciences); mouse anti-human Ki67 antigen (1/50; M7240, DakoCytomation). The Alexa fluor secondary antibodies were purchased from Thermo Fisher Scientific (1:200).

### Immunoblotting

For Western blot, cells were lysed in RIPA buffer with anti-protease and PhosSTOP tablets (Roche) and sonicated as previously described^50^. Equal amounts of protein (20 μg) were resolved in a 4–12% Bis–Tris gel and transferred to a membrane using an iBLOT2 Dry Blotting System (Thermo Fisher Scientific). Membranes were immunoblotted with the following antibodies: mouse anti-SV40T (1/50; DP-02, Calbiochem Merck Biosciences), Phospho-Ser133 CREB (1:1,000; Cell Signaling Technology), CREB (1:1,000; Cell Signaling Technology), β-actin (1:2,000; Sigma), alpha-Tubulin (1:2,000; Sigma). Species-specific HRP-linked secondary antibodies (Cell Signaling Technology) were used for detection and visualization was performed on an ImageQuant LAS 4,000 following ECL exposure (GE Healthcare).

### Statistics

Data were analyzed using GraphPad Prism 6 software and are presented as the mean ± SEM. Quantitative data are presented as the mean ± SEM. The number of experiments is indicated in the figure legends. Statistical significance was estimated using a 2-tailed Student’s *t* test. A *P* value less than 0.05 was considered significant.

### Data and resource availability

The datasets generated during and/or analyzed during the current study are available from the corresponding authors upon reasonable request. Most of the resources used during these studies are commercially available.

## Supplementary information

Supplementary Information
