# Supplementary material for: Regulated expression and function of the GABAB receptor in human pancreatic beta cell line and islets
Source: Sci Rep. 2020 Aug 10;10:13469. doi: 10.1038/s41598-020-69758-6 (PMC7417582; doi:10.1038/s41598-020-69758-6)
Supplement: Supplementary file 1 — Supplementary Information [file 41598_2020_69758_MOESM1_ESM.pdf]

Regulated expression and function of the $GABA_B$ receptor in human pancreatic beta cell line and islets.

Latif Rachdi[1]*, Alicia Maugein[1], Severine Pechberty[1], Mathieu Armanet[2], Juliette Hamroune[1], Philippe Ravassard[3], Stefano Marullo[1], Olivier Albagli[1], Raphael Scharfmann[1].

[1] Université de Paris, Institut Cochin, INSERM U1016, CNRS UMR 8104, 75014 Paris, France.

[2] Assistance Publique Hôpitaux de Paris, Cell Therapy Unit, Saint Louis Hospital, 75010 Paris, France.

[3] Institut du Cerveau et de la Moelle épinière (ICM), INSERM U1127, CNRS UMR 7225, Sorbonne Universités, 75013 Paris, France.

* corresponding authors

Institut Cochin, INSERM U1016, 123 bd du Port-Royal, 75014 Paris, France.
Phone: 33.1.76535569, Email : latif.rachdi@inserm.fr
ORCID iD : https://orcid.org/0000-0002-1089-2837

Table 1: Checklist for reporting human islet preparations used in research

Islet preparation	1	2	3	4	5	6	7	8[a]
**MANDATORY INFORMATION**								
Unique identifier	HSL P121	HSL P126	HSL P132	HSL P134	HSL P135	HSL P137	HSL P138	
Donor age (years)	57	61	53	57	63	55	49	
Donor sex (M/F)	F	M	F	F	M	M	F	
Donor BMI (kg/m^2)	23.4	23.8	18.4	24	28.4	30.5	27.7	
Donor HbA_{1c} or other measure of blood glucose control	NT	NT	NT	NT	NT	NT	NT	
Origin/source of islets[b]	APHP/hospital St Louis	APHP/hospital St Louis	APHP/hospital St Louis	APHP/hospital St Louis	APHP/hospital St Louis	APHP/hospital St Louis	APHP/hospital St Louis	
Islet isolation centre	hospital St Louis, Paris	hospital St Louis, Paris	hospital St Louis, Paris	hospital St Louis, Paris	hospital St Louis, Paris	hospital St Louis, Paris	hospital St Louis, Paris	
Donor history of diabetes? Please select yes/no from drop down list	No	No	No	No	No	No	No	
**If Yes, complete the next two lines if this information is available**								
Diabetes duration (years)								
Glucose-lowering therapy at time of death[c]								
**RECOMMENDED INFORMATION**								
Donor cause of death	Vascular	Vascular	Vascular	Vascular	Trauma	Vascular	Vascular	
Warm ischaemia time (h)	08:09	04:10	09:40	04:55	09:00	07:45	09:07	
Cold ischaemia time (h)								
Estimated purity (%)	70	70	70	70	70	80	70	
Estimated viability (%)	NT	NT	NT	NT	NT	NT	NT	
Total culture time (h)[d]	168	168	168	168	168	168	84+48h	
Glucose-stimulated insulin secretion or other functional measurement[e]	No	No	No	Yes	Yes	Yes	Yes	
Handpicked to purity? Please select yes/no from drop down list	Yes	Yes	Yes	Yes	Yes	Yes	Yes	
Additional notes	Fig. 1, 4A, 4B, 4C S6	Fig. 1, 4A, 4B, 4C, S6	Fig. 1, 4A, 4B, 4C, S6	Fig. 1, 4A, 4B, S6	Fig. 1, 5B, S6	Fig. 1, 5B, S6	Fig. 1, 5B	

Supplemental Table 2

Gene / Tube Name	5'Fw	3'Rv
CDKN1A	CCAAACACCTTCCAGCTCCTGTA	TGGAGAAACGGGAACCAGGACA
Cyclophilin-A	ATGGCAAATGCTGGACCCAACA	ACATGCTTGCCATCCAACCACT
GABBR1	AGCTGGGAAGAACATGCTATC	CAGAGGTATGAGAGACCCAAATC
GABBR2	ACCGGAATCAGAAGCTCATAAA	GGATCCATCAAGGCCAAAGA
IAPP	TTGGTGCCATTCTCTCATCTAC	CAAGTAATTCAGTGGCTCTCTCT
IGFBP3	CAGCCAGCGCTACAAAGTTG	GTCTTCCATTTCTCTACGGC
LINC00473	CAGCATACTTTGGCGGACCT	CCCATAGAGCGCTCCTTTCT
MAFA	ATTCTGGAGAGCGAGAAGTGCCAA	CGCCAGCTTCTCGTATTTCTCCTT
PAX4	AATTCCCTGGACTCAGGACTGCTT	TTCCAAGCCATACAGTAGTGGGCA
PCSK1	CATTCTTTGCCTGGTGCACTGTGT	TTGTGGCTGAGAAAGGAGACAGGT

Supplementary legends

Figure S1: *In vivo* expansion of SV40T-expressing cells in transplanted human neonatal pancreas.

Fragments of a human neonatal pancreas were transduced with lentiviral vectors expressing SV40T and hTERT under the control of the rat insulin2 promoter. They were transplanted under the kidney (K) capsule of SCID mice. Immunostainings for insulin (INS), SV40T and Ki67 were performed 3- and 7-months following transplantation. Scale bars: 100µm.

Figure S2: Immunofluorescence analysis of ECN90 cells.

(A) Morphology of Ecn90 cells at passage 69. **(B-D)** Ecn90 cells stained positive for insulin (INS), PDX1, SV40T, and expressed Ki67. Nuclei were stained with Hoechst 33342 fluorescent stain (blue). Scale bars: 12.5µm.

Figure S3: Decreased SV40T levels improves beta cell differentiation in ECN90.

Ecn90 cells were transfected with control nontarget siRNA (siControl) or siRNA targeting SV40T (siSV40T). **(A)** Western blot analyses performed 1- and 3-days following transfection (representative of n=3) **(B)** Immunofluorescence analysis of insulin (red) and SV40T (green) at day 6 following transfection. Nuclei were stained with Hoechst 33342 fluorescent stain (blue). Scale bars: 25µm. **(C)** Insulin content at day 6 following transfection. Results are shown as mean ± SEM (n = 3). ****P < 0.001 relative to control by Student's t test.

Figure S4: GABBR2 expression and activity in ECN90 does not depend on SV40T expression.

Ecn90 cells were transfected with control nontarget siRNA (siControl) or siRNA targeting SV40T (siSV40T). **(A) RT-**qPCR analyses of *GABBR1* and *GABBR2* (at day 6). *IAPP* and *CDKN1A* were used as control of siSV40T efficacy. **(B)** At day 5 post transfection, cells were pretreated with or without Baclofen (BAC) during 16h and next pulsed for 1h with or without Forskolin (FSK). RT-qPCR analyses indicate that BAC treatment blunts the induction by FSK of *MAFA* in control, but also in siSV40T transfected cells. Data are shown as the mean ± SEM (n = 3). *P < 0.05; ***P < 0.005; NS= not significant relative to control by Student's *t* test.

Figure S5: Baclofen does not signal in EndoC-βH1 cells and human islets.

EndoC-βH1 **(A, B)** and human islets **(C, D)** were pretreated with or without Baclofen during 16h and next pulsed for 1h with Forskolin (FSK). RT-qPCR analyses indicate that Baclofen treatment does not blunt the induction by FSK of *MAFA* and *PCSK1*. Data are shown as the mean ± SEM (n = 3-6). NS= not significant relative to control by Student's *t* test.

Figure S1

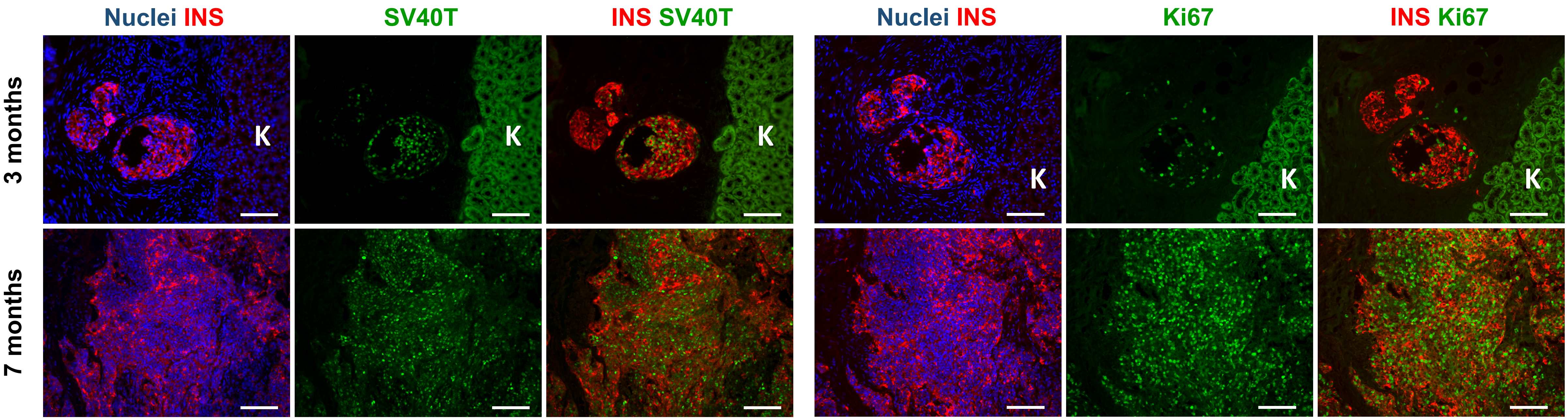

Nuclei INS
SV40T
INS SV40T
Nuclei INS
Ki67
INS Ki67
3 months
7 months
K

Figure S2

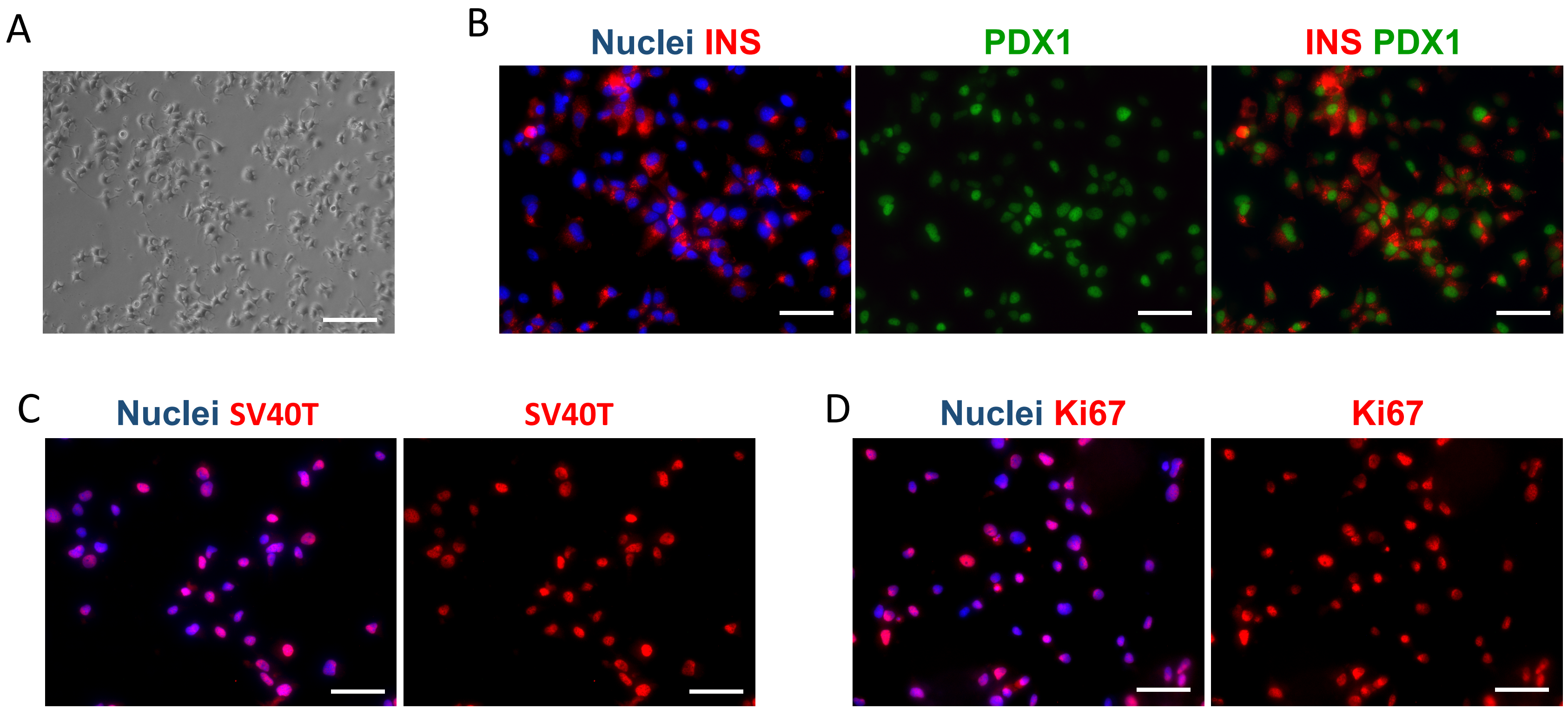
A
B
Nuclei INS
PDX1
INS PDX1
C
Nuclei SV40T
SV40T
D
Nuclei Ki67
Ki67

Figure S3

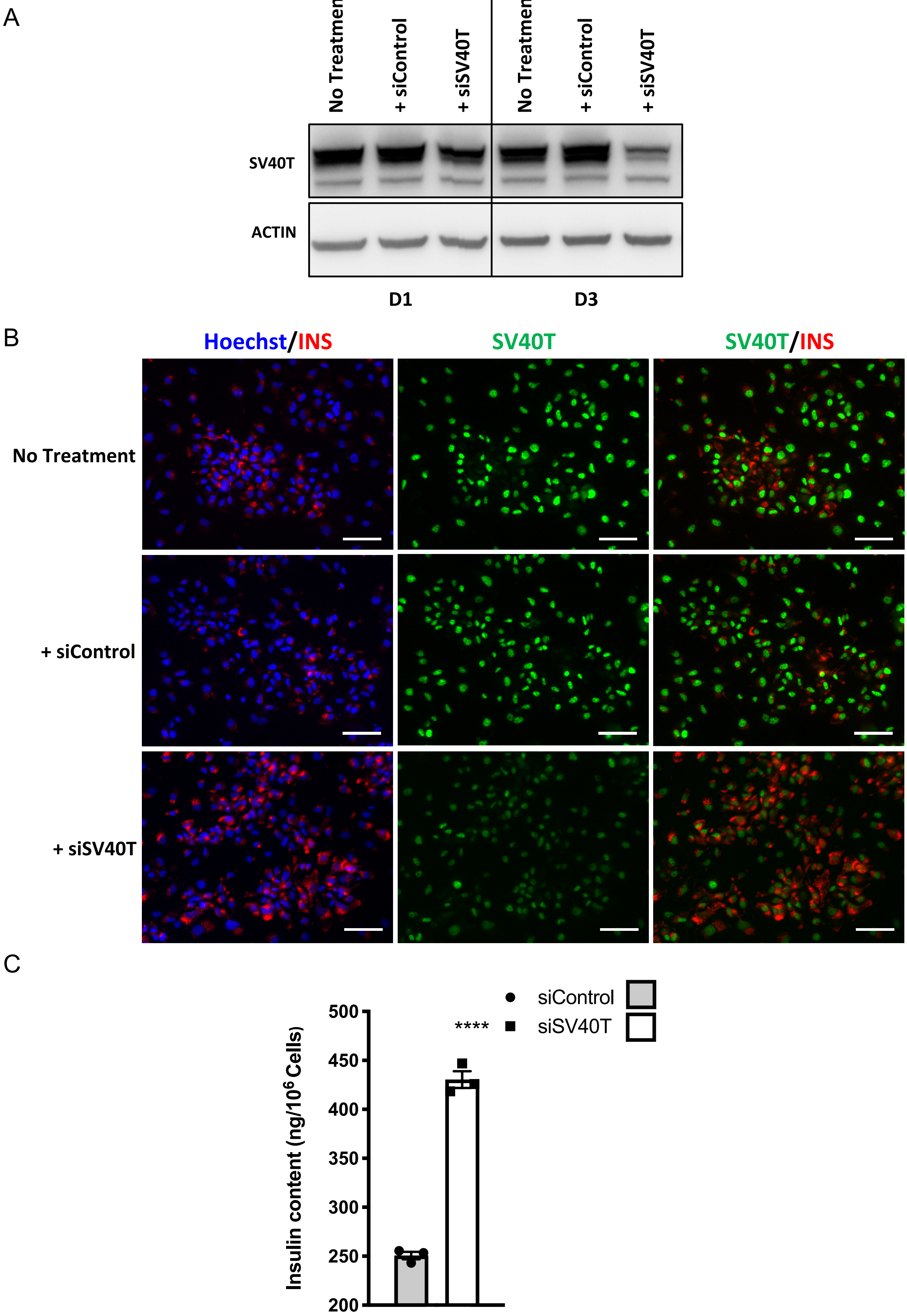
A
No Treatment
+ siControl
+ siSV40T
No Treatment
+ siControl
+ siSV40T
SV40T
ACTIN
D1
D3
B
Hoechst/INS
SV40T
SV40T/INS
No Treatment
+ siControl
+ siSV40T
C
siControl
siSV40T
****
Insulin content (ng/10^6 Cells)
500
450
400
350
300
250
200

Figure S4

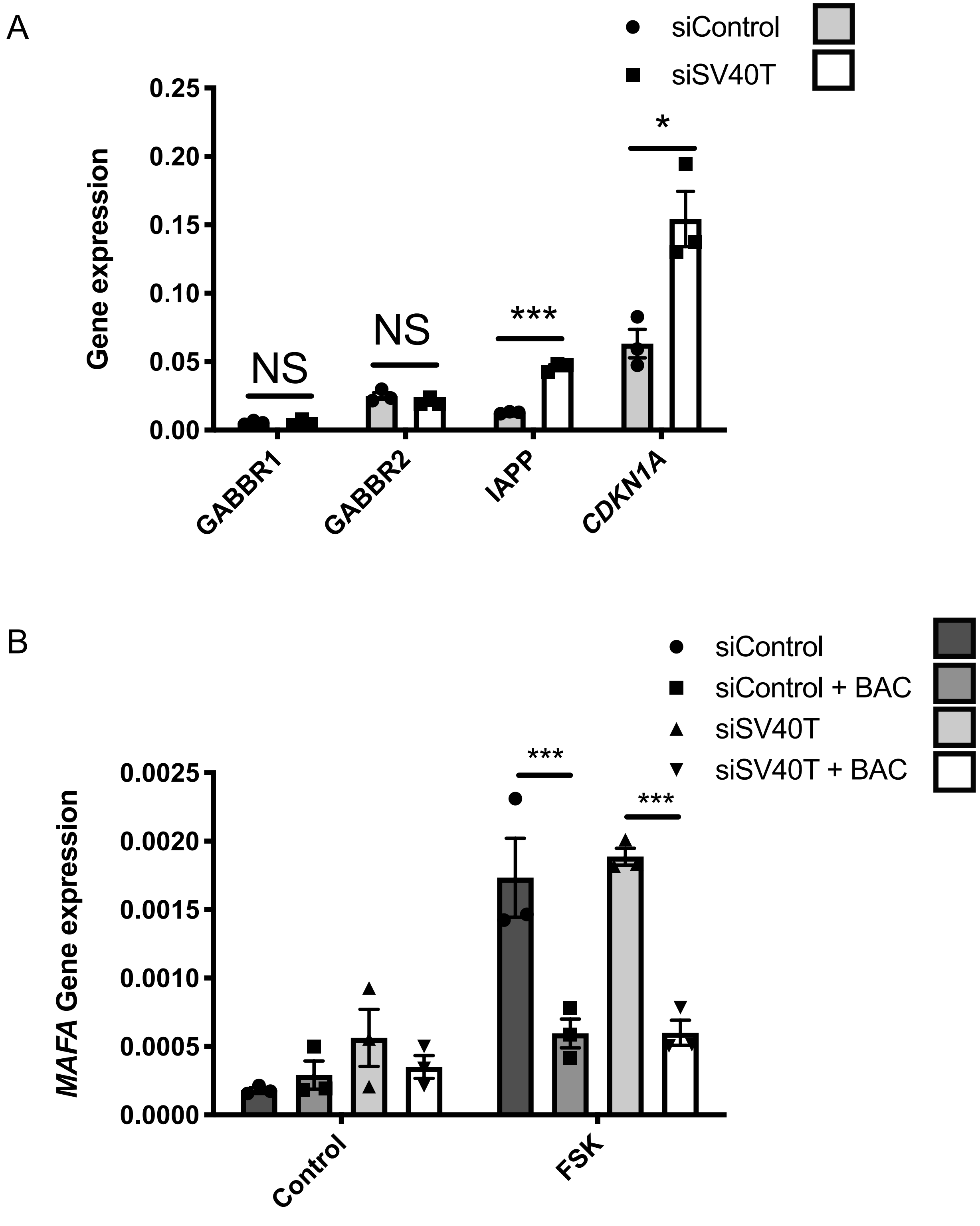
A
siControl
siSV40T
Gene expression
0.25
0.20
0.15
0.10
0.05
0.00
NS
NS
***
*
GABBR1
GABBR2
IAPP
CDKN1A
B
siControl
siControl + BAC
siSV40T
siSV40T + BAC
MAFA Gene expression
0.0025
0.0020
0.0015
0.0010
0.0005
0.0000
***
***
Control
FSK

Figure S5

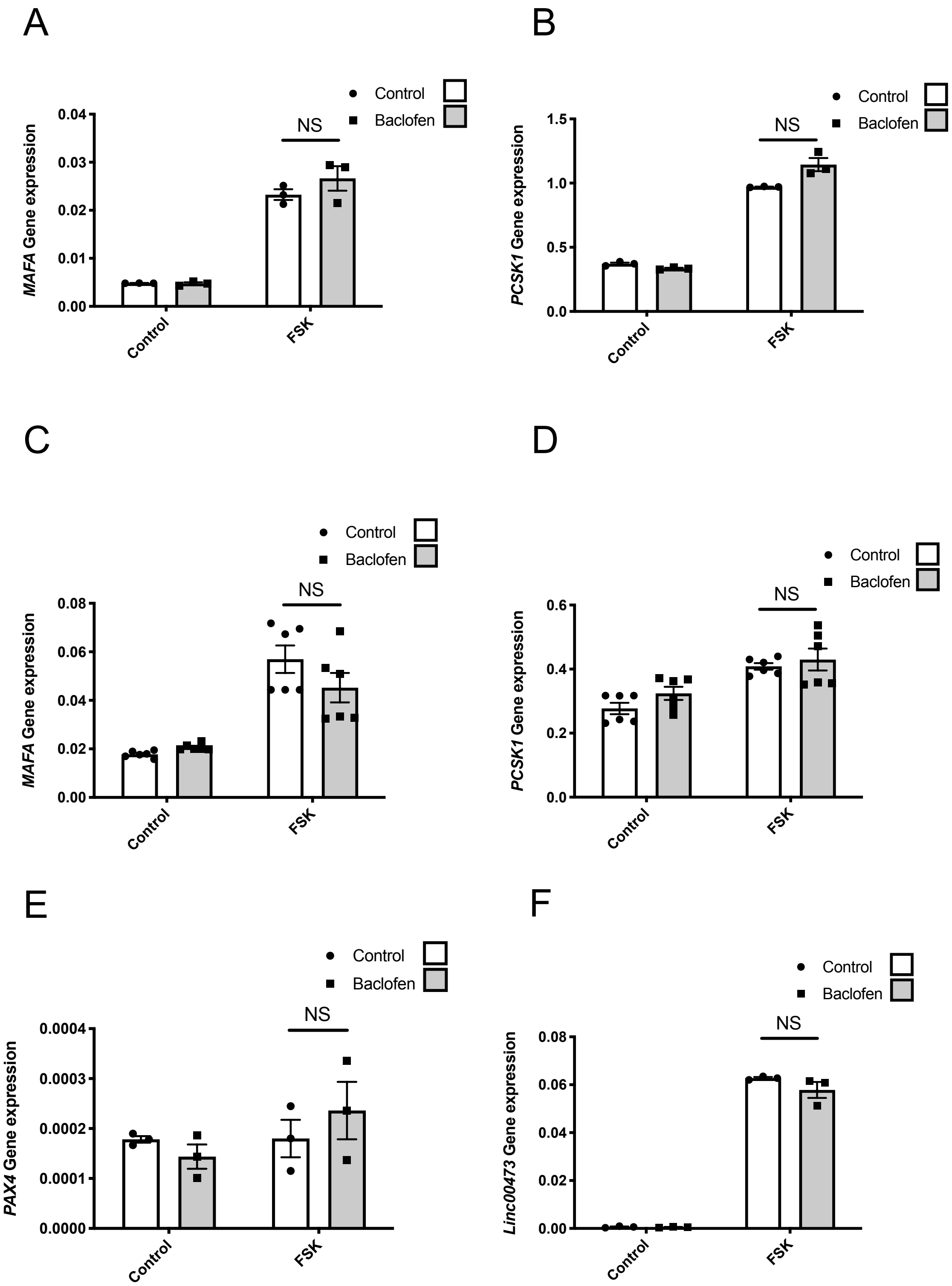
A
Control
Baclofen
NS
MAFA Gene expression
0.04
0.03
0.02
0.01
0.00
Control
FSK
B
Control
Baclofen
NS
PCSK1 Gene expression
1.5
1.0
0.5
0.0
Control
FSK
C
Control
Baclofen
NS
MAFA Gene expression
0.08
0.06
0.04
0.02
0.00
Control
FSK
D
Control
Baclofen
NS
PCSK1 Gene expression
0.6
0.4
0.2
0.0
Control
FSK
E
Control
Baclofen
NS
PAX4 Gene expression
0.0004
0.0003
0.0002
0.0001
0.0000
Control
FSK
F
Control
Baclofen
NS
Linc00473 Gene expression
0.08
0.06
0.04
0.02
0.00
Control
FSK
